# Remote ischaemic post conditioning in NE: A feasible and simple paradigm for self-protection in the brain?

**DOI:** 10.1038/s41390-025-03806-7

**Published:** 2025-01-21

**Authors:** Nicola J. Robertson, Raymand Pang, Derek J. Hausenloy

**Affiliations:** 1 Institute for Women’s Health, University College London, London, UK; 2 Centre for Clinical Brain Sciences, University of Edinburgh, Edinburgh, UK; 3 The Hatter Cardiovascular Institute, University College London, London, UK; 4 National Heart Research Institute Singapore, National Heart Centre, Singapore, Singapore; 5 Cardiovascular & Metabolic Disorders Program, Duke-National University of Singapore Medical School, Singapore, Singapore; 6 Yong Loo Lin School of Medicine, National University Singapore, Singapore, Singapore

Remote ischaemic post conditioning (RIPostC) has significant potential as a simple, non-invasive and effective treatment to reduce neurodevelopmental impairment (NDI) in babies with moderate-severe hypoxic ischaemic encephalopathy (HIE). In the recent publication by Lo and colleagues, a prospective, safety and dose escalation study of RIPostC is described in 32 babies with HIE undergoing hypothermia (HT).^1^ All patients received the designated RIPostC therapy without interruption or delay. There were no adverse events related to pain, vascular, cutaneous, muscular or neural safety effects. This study is an important step towards using RIPostC as a potential therapeutic intervention for babies with HIE, establishing the critical safety and feasibility data necessary for the design of future studies to investigate the potential efficacy of RIPostC to reduce NDI. However, before phase II clinical trials of RIPostC are considered in HIE, there is much to learn about the optimal RIPostC protocol or “RIPostC dose” from other conditions and from preclinical models.

While therapeutic HT improves survival without disability after moderate-severe HIE and remains the cornerstone treatment for suspected HIE, it is concerning that not all babies are protected in high-income settings^2^ and that HT is not beneficial in many low- and mid- income countries (LMIC).^3,4^ Clearly other therapies are needed to both augment HT and provide an alternative treatment to HT for HIE. Unlike other potential therapies for HIE which may require cold chain storage (e.g. erythropoietin, stem cells) or intravenous access (e.g. melatonin, caffeine, azithromycin), the RIPostC intervention uses readily available neonatal equipment (an appropriately sized blood pressure cuff), the intervention may be administered by medical or nursing staff, and it can be readily implemented in LMIC.

RIPostC is a manoeuvre that creates transient cycles of ischemia and reperfusion (I-R) to a remote site (e.g. limb).^5^ The goal is to protect the organ exposed to ischaemia, such as the brain, from further injury by inducing non-lethal ischemia in the remote site. The typical RIPostC intervention consists of 5 cycles of 5 mins ischaemia and 5 mins reperfusion using a blood pressure cuff inflated to supra-systolic pressure. RIPostC evolved from ischaemic preconditioning, discovered in 1986 by Murry^6^ in the myocardium; subsequently Kitagawa described ischaemic tolerance in the brain.^7^

RIPostC likely exerts neuroprotection through several overlapping mechanisms involving neuronal, humoral, and systemic effects.^8,9^ In the neuronal pathway (Fig. 1), the activation of afferent nerves and the release of neuropeptides at the site of RIPostC act on the efferent nerves at the larger site of ischemia to offer protection.^8^ The humoral pathway involves the release of hydrophobic factors that travel from the site of RIC to the site of ischemia for protection.^8,9^ Adenosine, bradykinin-2, opioids, glucagon-like peptides and Stromal Cell Derived Factor-1a are potential mediators.^8,10,11^ The systemic pathway involves the modification of the immune system by suppression of proinflammatory genes encoding proteins involved in leucocyte chemotaxis, adhesion, migration, and exocytosis,^8^ as well promoting microglia/macrophage transfer from M1 to M2 phenotype.^12^

**Fig. 1:**
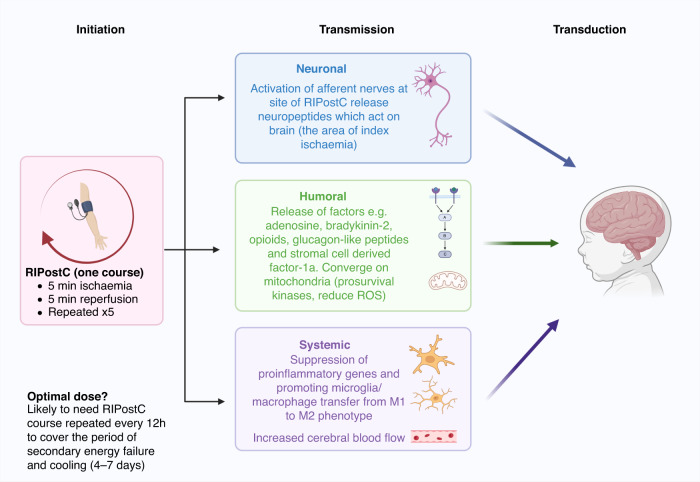
Possible mechanisms through which the RIPostC signal is initiated, transmitted and transduced into brain protection in babies with HIE. **Initiation:** The optimal dose of RIPostC (course and number of courses) is unknown although there is evidence that RIPostC needs to be repeated during the injury cascade for optimal benefit. This may span 4-7 days in the newborn with HIE to cover the period of secondary energy failure. **Transmission:** Transmission of the RIPostC signal is through neuronal, humoral and systemic pathways which converge on the mitochondria to reduced ROS and improve mitochondrial metabolism. RIPostC has been observed to increase cerebral blood flow. **Transduction:** These pathways combine to lead to potential beneficial effects on the index organ (brain) in HIE.

## Preclinical studies

There is strong preclinical support for the efficacy of RIPostC to improve neurological outcomes after cerebral ischaemia across multiple species. These studies suggest protection by RIPostC even with a delay of 3-6 h, although studies vary.^13,14^ The piglet model has been used to investigate RIPostC using magnetic resonance spectroscopy (MRS) biomarkers. In 2015, Robertson and colleagues reported robust protection of the white matter at 48 h with four 10 min cycles of bilateral lower limb I-R immediately after HI.^15^ Subsequent studies in the piglet have been less reassuring of RIPostC efficacy;^16,17^ this may be related to the several factors including “RIPostC dose and duration” as only one treatment cycle was administered with a 1 h RIPostC delay.

There is much that we can learn from other conditions to enhance the clinical translation of RIPostC for the treatment of HIE. RIPostC is gathering momentum as a potential therapy in adult stroke, traumatic brain injury (TBI) and necrotizing enterocolitis (NEC) in phase II and III clinical trials.

## Adult stroke

A recent Phase III clinical trial in adults with acute moderate ischaemic stroke (RICAMIS trial-including 1893 patients from 55 hospital in China) observed an improved chance of excellent outcome at 90 days with RIPostC administered after the ischaemic insult.^18^ The intensity, duration and patient selection of the RIPostC intervention is a vital factor; in the RICAMIS trial, treatment with RIPostC was performed *twice daily for 2 weeks within 48* *h of symptoms* as an adjunct to routine treatment, compared with routine treatment alone.^18^ The previously observed heterogenous effects of RIPostC are likely secondary to patient selection and RIPostC implementation.^18–20^ Other studies support the concept that a longer duration of RIPostC may exert a more neuroprotective effect. For example, RIPostC treatment twice daily for 2 weeks or until discharge with a mean duration of 11.2 days was neuroprotective.^19^

## Traumatic Brain Injury (TBI)

In a clinical trial of 84 patients with traumatic brain injury, a synergistic effect of head cooling and one course of RIPostC was observed with improved clinical outcomes compared to groups who received either separately.^21^ The authors also suggest that repeated courses of RIPostC might improve outcomes further.

## Necrotizing enterocolitis (NEC)

The clinical translation of RIPostC for NEC in preterm babies is inspirational. Preclinical studies in rat pups demonstrated that RIPostC improved blood flow to the intestine, reduced the intestinal damage of experimental NEC and prolonged survival.^22^ A recent Phase I safety study demonstrated that RIPostC (4 cycles of I-R repeated on two consecutive days early in the NEC course) was safe in preterm infants with NEC.^23^ A phase II feasibility RCT involving 12 centres in 6 countries is underway, to investigate the feasibility of RIPostC as a treatment for early-stage NEC in preterm neonates.

Considering the knowledge so far from clinical and preclinical studies in RIPostC, it is likely that the intervention needs to be “topped up” during the evolving phase of secondary energy failure after hypoxia ischaemia; hence repeated RIPostC courses (every 12–24 h) would be needed during the subacute phase (1st 4–7 days) in HIE for optimal benefit. It is also possible that longer-term RIPostC might ameliorate the continuing inflammatory dysregulation associated with tertiary brain injury during the weeks and months after HIE.^24^ For example, in adult stroke, a longer duration of RIPostC treatment (more than 300 days) was effective for secondary stroke prevention.^25^ There is also evidence that RIPostC performed on increased muscle mass leads to improved outcomes, suggesting that RIPostC in two limbs would be better than RIPostC in one limb.^26^

## Opportunities and challenges of RIPostC for treatment of HIE

RIPostC triggers endogenous protective pathways in distant organs such as the kidney, heart and brain, and represents an exciting new paradigm in neonatal neuroprotection. It is imperative that preclinical studies urgently define the optimal “RIPostC dose” after HI and that sensitive and specific biomarkers of RIPostC are discovered and validated. This will inform the design of future clinical trials in babies with HIE and ensure that the full potential of endogenous brain protection from RIPostC can be exploited. If RIPostC were to reduce NDI in HIE in clinical trials (with and without HT), this treatment could be rolled out with relative ease and lead to significant widespread benefit.

## References

[1] Lo, E. et al. Remote ischemic post-conditioning for neonatal encephalopathy: a safety and feasibility trial. *Pediatr. Res.*10.1038/s41390-024-03625-2 (2024). 10.1038/s41390-024-03625-2 39396091

[2] Schreglmann, M., Ground, A., Vollmer, B. & Johnson, M. J. Systematic review: long-term cognitive and behavioural outcomes of neonatal hypoxic-ischaemic encephalopathy in children without cerebral palsy. *Acta Paediatr.* **109**, 20–30 (2020). 31002422 10.1111/apa.14821

[3] Robertson, N. J. et al. Therapeutic hypothermia for birth asphyxia in low-resource settings: a pilot randomised controlled trial. *Lancet* **372**, 801–803 (2008). 18774411 10.1016/S0140-6736(08)61329-X

[4] Thayyil, S. et al. HELIX consortium. Hypothermia for moderate or severe neonatal encephalopathy in low-income and middle-income countries (HELIX): a randomised controlled trial in India, Sri Lanka, and Bangladesh. *Lancet Glob. Health* **9**, e1273–e1285 (2021). 34358491 10.1016/S2214-109X(21)00264-3 PMC8371331

[5] Lu, M., Wang, Y., Ren, H., Yin, X. & Li, H. Research progress on the mechanism of action and clinical application of remote ischemic post-conditioning for acute ischemic stroke. *Clin. Neurol. Neurosurg.* **244**, 108397 (2024). 38968813 10.1016/j.clineuro.2024.108397

[6] Murry, C. E., Jennings, R. B. & Reimer, K. A. Preconditioning with ischemia: a delay of lethal cell injury in ischemic myocardium. *Circulation* **74**, 1124–1136 (1986). 3769170 10.1161/01.cir.74.5.1124

[7] Kitagawa, K. et al. Ischemic tolerance’ phenomenon found in the brain. *Brain Res* **528**, 21–24 (1990). 2245337 10.1016/0006-8993(90)90189-i

[8] Lim, S. Y. & Hausenloy, D. J. Remote ischemic conditioning: from bench to bedside. *Front Physiol.* **3**, 27 (2012). 22363297 10.3389/fphys.2012.00027 PMC3282534

[9] Hess, D. C. et al. Remote ischaemic conditioning-a new paradigm of self-protection in the brain. *Nat. Rev. Neurol.* **11**, 698–710 (2015). 26585977 10.1038/nrneurol.2015.223

[10] Erbil, D. et al. GLP-1’s role in neuroprotection: a systematic review. *Brain Inj.* **33**, 734–819 (2019). 30938196 10.1080/02699052.2019.1587000

[11] Imitola, J. et al. Directed migration of neural stem cells to sites of CNS injury by the stromal cell-derived factor 1alpha/CXC chemokine receptor 4 pathway. *Proc. Natl Acad. Sci. USA* **101**, 18117–18122 (2004). 15608062 10.1073/pnas.0408258102 PMC536055

[12] Han, D. et al. Remote Limb Ischemic Postconditioning Protects against Ischemic Stroke via Modulating Microglia/Macrophage Polarization in Mice. *J. Immunol. Res*. **2021**, 6688053 (2021). 10.1155/2021/6688053 PMC7910075 33688509

[13] Zhou, Y. et al. Remote limb ischemic postconditioning protects against neonatal hypoxic ischemic brain injury in rat pups by the opioid receptor/Akt pathway. *Stroke* **42**, 439–444 (2011). 21183744 10.1161/STROKEAHA.110.592162 PMC3703505

[14] Ren, C. et al. Limb remote ischemic postconditioning protects against focal ischemia in rats. *Brain Res.* **1288**, 88–94 (2009). 19631625 10.1016/j.brainres.2009.07.029 PMC2744502

[15] Ezzati, M. et al. Immediate remote ischemic postconditioning after hypoxia ischemia in piglets protects cerebral white matter but not grey matter. *J. Cereb. Blood Flow. Metab.* **36**, 1396–1411 (2016). 26661194 10.1177/0271678X15608862 PMC4976661

[16] Kyng, K. J. Short-term outcomes of remote ischemic postconditioning 1 h after perinatal hypoxia-ischemia in term piglets. *Pediatr. Res.* **89**, 150–156 (2021). 32294662 10.1038/s41390-020-0878-6

[17] Andelius, T. C. K. et al. No added neuroprotective effect of remote ischemic postconditioning and therapeutic hypothermia after mild hypoxia-ischemia in a piglet model. *Front. Pediatr.* **26**, 299 (2020). 10.3389/fped.2020.00299 PMC7333529 32676486

[18] Chen, H. S. et al. RICAMIS investigators. effect of remote ischemic conditioning vs usual care on neurologic function in patients with acute moderate ischemic stroke: The RICAMIS randomized clinical trial. *JAMA* **328**, 627–636 (2022). 35972485 10.1001/jama.2022.13123 PMC9382441

[19] Zhao, W. et al. Remote ischaemic conditioning for preventing and treating ischaemic stroke. *Cochrane Database Syst. Rev.* **7**, CD012503 (2018). 29974450 10.1002/14651858.CD012503.pub2 PMC6513257

[20] England, T. J. et al. RECAST (Remote Ischemic Conditioning After Stroke Trial): A pilot randomized placebo controlled Phase II trial in acute ischemic stroke. *Stroke* **48**, 1412–1415 (2017). 28265014 10.1161/STROKEAHA.116.016429

[21] Hodoodi, F. et al. The effect of head cooling and remote ischemic conditioning on patients with traumatic brain injury. *iScience* **24**, 102472 (2021). 34169235 10.1016/j.isci.2021.102472 PMC8207229

[22] Ganji, N. et al. Remote ischemic conditioning in necrotizing enterocolitis. *Semin. Pediatr. Surg.* **32**, 151312 (2023). 37295298 10.1016/j.sempedsurg.2023.151312

[23] Zozaya, C. et al. Remote ischaemic conditioning in necrotising enterocolitis: a phase I feasibility and safety study. *Arch. Dis. Child Fetal Neonatal Ed.* **108**, 69–76 (2023). 35940871 10.1136/archdischild-2022-324174 PMC9763186

[24] Fleiss, B. & Gressens, P. Tertiary mechanisms of brain damage: a new hope for treatment of cerebral palsy? *Lancet Neurol.* **11**, 556–566 (2016). 10.1016/S1474-4422(12)70058-3 22608669

[25] Li, Q. et al. Remote ischemic conditioning with medical management or reperfusion therapy for acute ischemic stroke: a systematic review and meta-analysis. *Neurology* **102**, e207983 (2024). 38457772 10.1212/WNL.0000000000207983 PMC11033986

[26] Kharbanda, R. K. et al. Transient limb ischemia induces remote ischemic preconditioning in vivo. *Circulation* **106**, 2881–2883 (2002). 12460865 10.1161/01.cir.0000043806.51912.9b

